# Utility of intraoperative real-time near-infrared fluorescence surgery for spinal schwannoma

**DOI:** 10.3171/2021.10.FOCVID21158

**Published:** 2022-01-01

**Authors:** Jun Muto, Yutaka Mine, Sota Nagai, Naoyuki Shizu, Hiroki Takeda, Daiki Ikeda, Akifumi Saito, Masahiro Joko, Mitsuhiro Hasegawa, Shinjiro Kaneko, Tatsushi Inoue, John Y. K. Lee, Yuichi Hirose

**Affiliations:** 1 Department of Neurosurgery, Fujita Health University, Toyoake;; 2 Department of Spine and Spinal Cord Surgery, Fujita Health University, Toyoake;; 3 Department of Orthopaedic Surgery, Fujita Health University, Toyoake;; 4 Department of Neurosurgery, Brain Nerve Center, Saiseikai Yokohamashi Tobu Hospital, Yokohama, Japan; and; 5 Department of Neurosurgery, University of Pennsylvania, Philadelphia, Pennsylvania

**Keywords:** delayed window indocyanine green, near-infrared spectroscopy, spinal tumors

## Abstract

The authors report the first cases of fluorescence-guided spinal surgery of schwannomas using near-infrared fluorescence imaging with the delayed window indocyanine (ICG) green (DWIG) technique for accurate real-time intraoperative tumor visualization.

Patients with intradural spinal schwannomas received 0.5 mg/kg ICG at the beginning of surgery. After 1 hour, using the DWIG technique, near-infrared spectroscopy (NIRS) detected the spinal schwannomas, showing the exact tumor location and boundaries. DWIG with NIRS microscopy confirmed the exact location of spinal schwannomas before and after opening of the dura mater, thereby facilitating successful tumor dissection from the surrounding tissues, tumor resection, and confirmation of tumor removal.

The video can be found here: https://stream.cadmore.media/r10.3171/2021.10.FOCVID21158

## Transcript

This video demonstrates intraoperative real-time near-infrared fluorescence surgery for spinal schwannoma using indocyanine green. We present two representative cases.

### 0:32 Methods.

The second window indocyanine green (SWIG) technique seems to facilitate brain tumor removal and was used for gliomas,^1^ meningiomas,^2^ and metastatic tumors.^3,4^ SWIG protocols by Madajewski et al.^5^ and Lee et al.^1–3^ administered 5.0 mg/kg ICG 24 hours before surgery. In contrast, we modified this technique using the delayed ICG window, administering 0.5 mg/kg ICG at the beginning of surgery. Then, the surgeons operated with the guidance of tumor fluorescence after more than 1 hour. KINEVO 900 and Pentero [Carl Zeiss Co., Ltd.] microscopes were used for near-infrared (NIR) imaging. Nerve stimulator was used to determine if the adherent structure was a function nerve root.

The fluorescence parameters are relative, not absolute; therefore, we obtained a background reading from the adjacent healthy spinal cord to determine the signal-to-background ratio (SBR).

### 1:38 Case Presentation.

A 55-year-old female with neurofibromatosis type 2 presented with neck pain. A spinal Gd-MRI revealed a well-circumscribed, heterogeneous intradural extraaxial mass in the upper cervical spine. Neurologically, the patient was intact, with no motor and sensory disturbances. She had multiple intracranial meningiomas and had surgeries for tumor removal twice before.

### 2:08 Preoperative Imaging Studies.

MRI with contrast demonstrated a 25-mm intradural, extramedullary cystic tumor at the C1–2 level.

### 2:20 Surgery.

The skin incision was performed from the inion to C5. The nuchal ligament was incised, and the spinous processes of C1–3 were identified. The C2 posterior arch was divided, preserving the muscles surrounding the spinous processes. Right C1–2 hemilaminectomy was performed, with a 15-mm bone window. ICG was administered with a 0.5-mg/kg dose at the beginning of surgery.

After the C1–2 hemilaminectomy, the dura was exposed. On preoperative MRI, we suspected meningioma. A strong NIR signal could be detected through the dura mater more than 1 hour after injection, and the tumor was visualized clearly. Its fluorescence moved synchronously with respiration, suggesting a diagnosis of schwannoma rather than meningioma. We changed the dural incision at the midline because we suspected the schwannoma by NIRS findings.

After the midline dural incision, the tumor capsule was exposed. The arrow shows the origin of the tumor, the posterior root of C3. The boundary was clearly visible, and the positivity for ICG fluorescence on NIRS helped identify the tumor margins and differentiate the mass from the surrounding tissues.

NIRS can help to decide the cutting point from the root. White arrow shows the nerve of tumor origin. Cutting the root from the tumor. After the rumor resection, NIR shows the fluorescence remaining ICG inside the tumor.

No residual tumor on the light field and no NIR signal were observed after the resection. The dura mater was closed watertightly. Each half of the C2 split spinous process was reapproximated using a strong suture. Muscles were sutured layer by layer.

### 4:32 Postoperative Imaging Studies.

Postoperative Gd-MRI showed no enhanced lesions and confirmed the complete resection. After the surgery, no new neurological deficits were found. The final pathology identified the tumor as a schwannoma. Relative tumor fluorescence to spinal parenchyma, SBR was 3.04.

### 4:58 Second Case Presentation.

A 79-year-old male presented with right lower extremity weakness. Neurological findings showed right motor weakness in the lower extremity and sensory disturbance below T7. Past medical history indicated hypertension.

### 5:16 Preoperative Imaging Studies.

The MRI revealed a well-circumscribed, 38-mm mass with heterogeneous enhancement between C6 and T2.

### 5:29 Surgery.

ICG was administered with a 0.5-mg/kg dose at the beginning of surgery. After the laminectomy from C6 to T2, the dura mater was exposed, and a weak fluorescence could be observed through the dura, more than 1 hour after injection. The microscope (Pentero) and NIRS accurately localized the intradural extramedullary tumor after incising the dura. Once exposed, the tumor was bluntly dissected in proximity to the capsule to separate the mass from the surrounding structures. In patients where the nerve roots appeared to adhere to the capsule, a nerve stimulator can be used to determine if the adherent structure is a functional motor nerve root. NIRS can help to identify the exact tumor location during the dissection. Proximal and distal root connections were dissected for complete tumor removal. There was no postoperative residual tumor and no NIR signal after tumor resection, suggesting total tumor removal. NIR showed the fluorescence remaining ICG inside the tumor.

### 6:40 Postoperative Imaging Studies.

Postoperative Gd-MRI showed no evidence of residual tumors. After the surgery, no new neurological deficits were found. The final pathology identified the tumor as a schwannoma. Relative tumor fluorescence to spinal parenchyma, SBR was 2.79.

### 7:02 Conclusions.

NIRS of ICG allowed stronger fluorescence from the tumor relative to normal spine parenchyma. Merits of fluorescence-guided spinal surgery were identification of real-time intraoperative tumor localization, confirmation of adequate laminectomy, differentiation from the tumor to surrounding tissues, confirmation of residual tumor. The delayed window ICG (DWIG) technique is not indispensable, but helpful for spinal tumor surgery.

## Disclosures

Funding for this study was provided by the Japan Research Foundation for Clinical Pharmacology (to Dr. Muto).

Dr. Lee reported other from VisionSense–Medtronic Elevision during the conduct of the study and has previously owned stock options in VisionSense. Dr. Hirose reported receiving grants from Eisai, Daiichi-Sankyo, and Chugai Pharmaceuticals, outside the submitted work.

## Author Contributions

Primary surgeon: Muto, Kaneko, Inoue. Assistant surgeon: Nagai, Takeda, Ikeda, Saito, Kaneko. Editing and drafting the video and abstract: Muto, Mine, Shizu, Kaneko. Critically revising the work: Muto, Mine, Joko, Hasegawa, Kaneko, Hirose. Reviewed submitted version of the work: Muto, Mine, Hasegawa, Kaneko, Lee, Hirose. Approved the final version of the work on behalf of all authors: Muto. Supervision: Mine, Hirose. Conception and design: Muto, Mine.

## Supplemental Information

### Ethics Approval and Clinical Trial

This prospective study was approved by our local Clinical Research Ethics Committee (CRB4180003). This trial was registered with the Japan Registry of Clinical Trials (RCTs 041190064).

### Patient Informed Consent

All enrolled patients agreed to and signed informed consent for this project.
